# Supplementary material for: Transcriptome-Wide Evaluation Characterization of microRNAs and Assessment of Their Functional Roles as Regulators of Diapause in Ostrinia furnacalis Larvae (Lepidoptera: Crambidae)
Source: Insects. 2024 Sep 14;15(9):702. doi: 10.3390/insects15090702 (PMC11432511; doi:10.3390/insects15090702)
Supplement: Supplementary file 1 [file insects-15-00702-s001.zip › Supplementary Material_Table S1.pdf]

Table. S1 Primer information used in qPCR

**Primers**	**Primer sequence（5'-3'）**
PC-3p-230_27417	F: TGCCGCGGCTCCGACCATTTCG
miR-306a-5p	F: CCAGGTACTAGGTGACTCTGA
miR-10485-5p	F: ACAGTCTACCCGGACAGGCCG
miR-2738	F: CTTAGAACGGCCATCTGATG
miR-2766	F: TCAGTCTTGTCGAATGGTGGGTAA
miR-277-3p	F: TAAATGCACTATCTGGTACGACAT
miR-277	F: TAAATGCACTATCTGGTACGACA
miR-2767	F: TAAGTAAATCTCGTGCGGCTTGTTTT
U6	F: GGAACGATACAGAGAAGATTAGC
	R: TGGAACGCTTCACGAATTTGCG
	R: Universal reverse Q primer[c](10μM）
