# Supplementary material for: Transcriptome-Wide Evaluation Characterization of microRNAs and Assessment of Their Functional Roles as Regulators of Diapause in Ostrinia furnacalis Larvae (Lepidoptera: Crambidae)
Source: Insects. 2024 Sep 14;15(9):702. doi: 10.3390/insects15090702 (PMC11432511; doi:10.3390/insects15090702)
Supplement: Supplementary file 1 [file insects-15-00702-s001.zip › Supplementary Material_Table S2.pdf]

Table. S2 Counts and frequency of different reads types in the three diapause states of *Ostrinia furnacalis*.

		ND		D		DT	
**lib**	**type**	**Total**	**% of Total**	**Total**	**% of Total**	**Total**	**% of Total**
Raw reads	NA	29,481,404	100.00	26,456,988	100.00	32,177,388	100.00
3ADT&length filter	Sequence type	7,354,659	24.95	6,028,111	22.78	9,935,688	30.88
Junk reads	Sequence type	67,901	0.23	68,120	0.26	59,748	0.18
Clean reads	Sequence type	22,058,844	74.82	20,360,757	76.96	22,181,952	68.94
