# Supplementary figures and images for: Transcriptome-Wide Evaluation Characterization of microRNAs and Assessment of Their Functional Roles as Regulators of Diapause in Ostrinia furnacalis Larvae (Lepidoptera: Crambidae)
Source: Insects. 2024 Sep 14;15(9):702. doi: 10.3390/insects15090702 (PMC11432511; doi:10.3390/insects15090702)

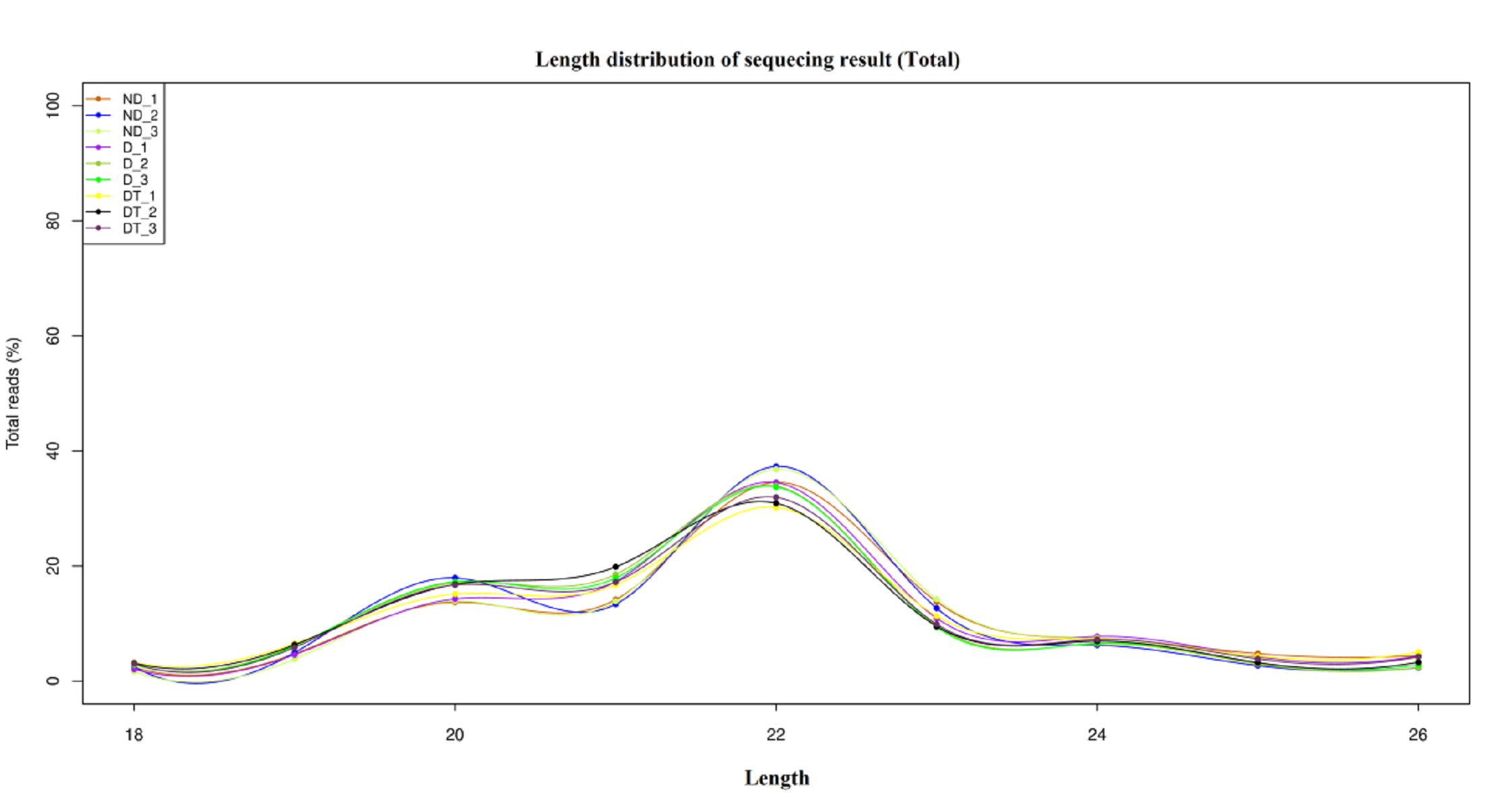

Supplement: Supplementary file 1 [file insects-15-00702-s001.zip › Supplementary Material_Fig. S1.JPG]

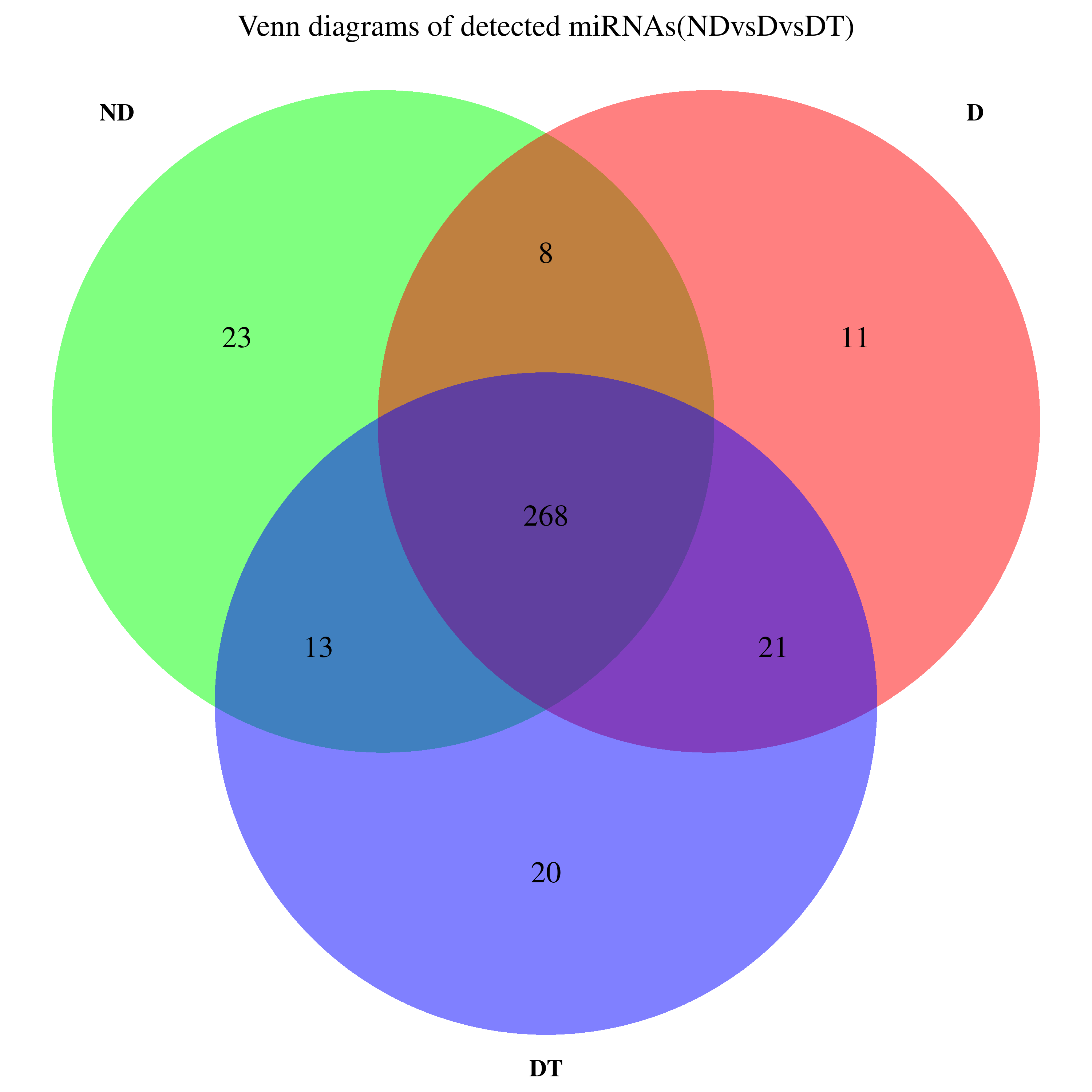

Supplement: Supplementary file 1 [file insects-15-00702-s001.zip › Supplementary Material_Fig. S2.png]
